# A Field-Deployable Reverse Transcription Recombinase Polymerase Amplification Assay for Rapid Detection of the Chikungunya Virus

**DOI:** 10.1371/journal.pntd.0004953

**Published:** 2016-09-29

**Authors:** Pranav Patel, Ahmed Abd El Wahed, Oumar Faye, Pauline Prüger, Marco Kaiser, Sasikanya Thaloengsok, Sukathida Ubol, Anavaj Sakuntabhai, Isabelle Leparc-Goffart, Frank T. Hufert, Amadou A. Sall, Manfred Weidmann, Matthias Niedrig

**Affiliations:** 1 Centre for Biological Threats and Special Pathogens, Robert Koch Institute, Berlin, Germany; 2 Division of Microbiology and Animal Hygiene, Georg-August-University, Goettingen, Germany; 3 Arbovirus Unit, Pasteur Institute, Dakar, Senegal; 4 GenExpress Gesellschaft für Proteindesign, Berlin, Germany; 5 Department of Microbiology, Faculty of Science, Mahidol University, Bangkok, Thailand; 6 Functional Genetics of Infectious Diseases Unit, Institute Pasteur, Paris, France; 7 French Armed Force Biomedical Research Institute (IRBA) Marseille, France; 8 Institute of Microbiology & Virology, Brandenburg Medical School Fontane, Senftenberg, Germany; 9 Institute of Aquaculture, University of Stirling, Stirling, Scotland, United Kingdom; Naval Medical Research Center, UNITED STATES

## Abstract

**Background:**

Chikungunya virus (CHIKV) is a mosquito-borne virus currently transmitted in about 60 countries. CHIKV causes acute flu-like symptoms and in many cases prolonged musculoskeletal and joint pain. Detection of the infection is mostly done using RT-RCR or ELISA, which are not suitable for point-of-care diagnosis.

**Methodology/Principal Findings:**

In this study, a reverse transcription recombinase polymerase amplification (RT-RPA) assay for the detection of the CHIKV was developed. The assay sensitivity, specificity, and cross-reactivity were tested. CHIKV RT-RPA assay detected down to 80 genome copies/reaction in a maximum of 15 minutes. It successfully identified 18 isolates representing the three CHIKV genotypes. No cross-reactivity was detected to other alphaviruses and arboviruses except O'nyong'nyong virus, which could be differentiated by a modified RPA primer pair. Seventy-eight samples were screened both by RT-RPA and real-time RT-PCR. The diagnostic sensitivity and specificity of the CHIKV RT-RPA assay were determined at 100%.

**Conclusions/Significance:**

The developed RT-RPA assay represents a promising method for the molecular detection of CHIKV at point of need.

## Author Summary

CHIKV is transmitted to humans via mosquitos. CHIKV induces clinical signs similar to Influenza, Dengue, and Zika viruses. We have developed a molecular assay for the detection of CHIKV genome based on isothermal„recombinase polymerase amplification (RPA) assay”performed at 42°C. The result was obtained in maximum of 15 minutes, which is 4–6 times faster than the current molecular diagnostic techniques. Our CHIKV RPA assay is rapid and sensitive, as well as easy to use at the point of need.

## Introduction

Chikungunya virus (CHIKV) is a mosquito-borne virus belonging to the genus alphavirus, family *Togaviridae* and spread by *Aedes* mosquitoes. CHIKV is the aetiological agent of chikungunya fever (CF) and was first isolated in 1952 from the serum of a febrile patient during an outbreak in Tanzania, Africa [1]. CHIKV infection is characterized by an abrupt onset of fever lasting two to five days, frequently accompanied by arthralgia. The disease is usually self-limiting, but joint pain symptoms can persists for weeks up to years [2–4]. Other common symptoms include muscle pain, headache, nausea, fatigue and rash similar to the dengue virus infection. Besides CHIKV, the *Alphavirus* genus includes viruses such as O'nyong'nyong virus (ONNV), Ross River virus (RRV), Barmah Forest virus (BFV), Sindbis virus (SNV) and Mayaro virus (MAYV) [5].

Since its discovery in Africa, CHIKV has repeatedly caused outbreaks in Africa, India, Southeast Asia, the Middle East and Europe with irregular intervals [6–10]. Phylogenetic analysis of CHIKV showed that the virus has evolved into three distinct genotypes: Asian, West African and Eastern/Central /South African (ECSA) [11]. A single-base mutation E1-A226V in a strain of the ECSA genotype enhanced replication of the virus in *Ae*. *albopictus*, and led to a large-scale epidemic on La Reunion in 2005 [12]. This ECSA genotype was subsequently associated with epidemics in the Indian Ocean region, and the Asian genotype has been associated with recent outbreaks in the Pacific region [13].

The first local transmission of CF in the Americas was reported from the Caribbean islands in December 2013. The local transmission of the disease has been reported in 45 countries or territories throughout world with more than 1.7 million suspected cases (CCDR October 20, 2015, http://www.cdc.gov/chikungunya/geo/index.html). It appears to have been introduced twice to Brazil, once from the Pacific region and once from Africa [14].

Current diagnosis of CHIKV is based on three main laboratory methods: virus isolation, reverse transcription-polymerase chain reaction (RT-PCR) and serological tests such as plaque reduction neutralizing test (PRNT), enzyme-linked immunosorbent assays (ELISA) or immunofluorescence test (IFT). Commonly, blood and serum samples are used as specimens for CHIKV diagnosis. Depending on the type of sample and the time of sample collection relative to symptoms (acute or convalescent phase of disease), an appropriate diagnostic method is applied to the samples [15].

A pronounced viraemia of up to 10^9^ viral genomes can be observed mainly on days 1–6 after onset of disease and in some cases for longer [16], therefore, virus isolation and RT-PCR are performed on acute phase specimens collected during the first week after onset of symptoms. Several RT-PCR assays have been published for the detection of CHIKV RNA in clinical specimens and mosquito samples [17–23]. Real-time RT-PCR based assays are suitable for clinical diagnosis due to the closed tube assay format, the option for quantification of viral load, high sensitivity and specificity. Serological tests are applied to either acute or convalescent phase samples for the detection of IgM and IgG anti-CHIKV antibodies. Serological diagnosis is confirmed by direct detection of IgM anti-CHIKV antibodies or by determining a four-fold increase in CHIKV-specific antibody titers in acute and convalescent samples by ELISA, IFT or PRNT tests [15].

Since the clinical picture of CHIKV is similar to that of DENV and Zika virus, a simple and rapid molecular assay would be needed to select the best treatment approach. The recombinase polymerase amplification (RPA) assay utilizes enzymes and proteins in order to allow the amplification of the DNA at a constant temperature (38–42°C) [24]. The presence of the amplicon is detected *via* the exo-probe, which include an internal abasic site mimic (tetrahydrofuran, THF) flanking fluorophore and Quencher as well as pathogen-specific 30 and 15 bases at both the 5´ and 3´ prime ends, respectively. Upon the hybridization of the exo-probe to the complementary sequence the Exonuclease III cleaves at the THF site and the fluorescence signal is generated. RPA assay was successfully developed for the detection of DENV and YFV as well as other human and veterinary pathogens [25–32]. Moreover, a mobile suitcase laboratory was established for allowing the deployment of the RPA in the field for identifying Ebola virus infected case [33].

In this study, we developed and evaluated a reverse-transcription recombinase polymerase amplification (RT-RPA) assay as potential point-of-care (POC) diagnostic tool for rapid detection of CHIKV. The RT-RPA assay was designed by targeting the non-structure protein 1 (nsP1). The sensitivity and specificity of the method was evaluated using strains of the three genotypes of CHIKV and compared to reference RT-PCR methods. Finally, the performance of the RT-RPA assay was evaluated on acute-phase serum samples for clinical diagnosis of CHIK.

## Methods

### Ethical Statement Clinical Samples

In total, the study included 78 patients with a history of sudden onset of fever, headache, fatigue, nausea, vomiting, rash, myalgia and severe and very painful polyarthralgia suggestive of CHIK infection. Fifty-eight plasma samples were provided by the Department of Microbiology, Faculty of Science, Mahidol University, Bangkok, Thailand (Ethical approval ID: COA. No. MU-IRB 2010/251.3108). Twenty CHIKV positive sera samples collected from patients suspected to be infected by CHIKV during routine medical examination, were provided by French National Reference Center for Arboviruses, Marseille, France. Patients had given oral consent according to the national ethical regulations. All used samples were handled anonymously.

### Virus Preparations

All the different CHIKV virus strains (Table 1) used in this study were derived from cell culture and kindly provided by the European Network for Diagnostics of Imported Viral Diseases (ENIVD); the Bernard-Nocht Institute in Hamburg, Germany; French National Reference Center for Arboviruses, Marseille, France and the Pasteur Institute of Dakar in Senegal. The Robert-Koch Institute, Berlin in Germany and the Pasteur Institute de Dakar in Senegal provided different *flavivviruses*, *alphaviruses* and Rift Valley Fever Virus (Table 2) for cross reactivity testing.

**Table 1:** List of the 18 isolates representing the three CHIKV genotypes. All isolates were screened with real-time RT-PCR assays and RT-RPA.

Name	Isolate origin/year	CHIKV clade	Real-time RT-PCR (Ct)	RT-RPA (Tt)
CHIKV 902/BHK	ind. Ocean 01/2007	ECSA	23.00	3.7
CHIKV 236/	ind. Ocean 01/2007	ECSA	22.50	3.7
CHIKV BHK/3162	ind. Isolate 01/2007	ECSA	24.69	4.0
CHIKV La Réunion 684–1	La Réunion /2006	ECSA	24.01	3.7
CHIKV African S27	East Africa	ECSA	21.47	3.7
CHIKV H20235	ST Martin/2013	Asia	16.33	3.7
CHIKV Ar D 93311	Senegal/1993	West African	17.76	2.7
CHIKV H D 103090	Senegal/1993	West African	20.65	3.3
CHIKV H D 103091	Senegal/1993	West African	21.58	3.3
CHIKV Ar A 30543	Côte d'Ivoire/1993	West African	19.67	5.3
CHIKV Ar A 30548	Côte d'Ivoire/1993	West African	19.22	3.3
CHIKV Ar D 122544	Senegal/1996	West African	24.49	2.7
CHIKV Ar D 128438	Senegal/1997	West African	22.39	2.7
CHIKV H D 131124	Senegal/1998	West African	20.27	3.0
CHIKV Ar D 156076	Senegal/2004	West African	18.93	3.3
CHIKV Ar D 175374	Senegal/2004	West African	20.89	3.0
CHIKV Ar D 175381	Senegal/2004	West African	23.43	3.0
CHIKV H D 180760	Senegal/2005	West African	Not detected	5.3

**Table 2:** Reactivity of *flaviviruses*, *phlebovirus* and *alphaviruses* in the CHIKV RT-RPA assay using two different primers pairs. The RF2+RR2 detected only the CHIKV, but not other viruses. While RF+RR3 detected both CHIKV and O'nyong'nyong virus.

Family	Name	Strain	RT-RPA assay	
			RF+RR3	RF2+RR2
***Flaviviridae***	Dengue virus serotype 1	VR344 (Thai 1958 strain)	-	-
	Dengue virus serotype 2	VR345 (TH-36 strain)	-	-
	Dengue virus serotype 3	VR216 (H87 strain)	-	-
	Dengue virus serotype 4	VR217 (H241 strain)	-	-
	West Nile Virus Israel (lineage 1)	H. Bin, Sheba Medical Center, Israel	-	-
	West Nile Virus (lineage 2)	B956/AY532665	-	-
	Yellow Fever Virus Asibi	AY640598.1	-	-
	Yellow Fever Virus 17D	17D RKI #142/94/1	-	-
	Zika Virus	MR766	-	-
	Japanse Encephalitis virus	ATCC SA14-14-2	-	-
***Bunyaviridae***	Rift Valley Fever Virus	ZH548	-	-
***Alphaviridae***	Ndumu virus	-	-	-
	Middelburg virus	-	-	-
	Babanki virus	-	-	-
	Zingilamo	An B 1245 a	-	-
	Semliki Forest	-	-	-
	Semliki Forest virus	M1V2 Turana	-	-
	Ross river fever virus	T 48/DQ226993	-	-
	Barmah Forest virus	BH2193/U73745	-	-
	Sindbis virus	Edgar 339	-	-
	Venezuelan equine encephalitis virus	VEEV 8–01	-	-
	O'nyong'nyong virus	An D SS 234	**+**	-

### RNA Extraction

Viral RNA was isolated from 140 μl aliquots of cell culture supernatants or serum sample, using the QIAamp Viral Mini Kit (QIAGEN, Hilden, Germany) according to the manufacturer's instructions. RNA was eluted in 60–100 μl of elution buffer and stored at -80°C until further use.

### Primer and Probe Design for RT-RPA

To identify the most conserved regions in the CHIKV genome for primer and probe design of the RPA assay, we first analyzed an alignment of fifty CHIKV full genome sequences from the RNA virus database (http://bioafrica.mrc.ac.za/rnavirusdb/) using GENEIOUS [34]. The nsP1 gene region from nucleotide position 131–306 nt was chosen for final RT-RPA assay design. In total, 196 Genbank CHIKV sequences containing the conserved nsP1 region were re-aligned using GENEIOUS. Based on the alignment, two forward primers, two reverse primers and a single probe (Table 3) covering all known variants of CHIKV sequences were selected.

**Table 3:** List of the oligonucleotides of the RT-RPA and real-time RT-PCR assays.

Assay format	Oligo name	Sequence 5´- 3´	Target region	Orientation	Position NC004162
RT-RPA	CHIKV RF2	TCA CAY CRA ATG ACC AYG CTA ATG CTA GAG C	nsP1	S	171–201
	CHIKV RR2	TTC CTR TCC GAC ATC ATC CTC CTT GCT GGY GC		AS	272–303
	CHIKV RF	TGC ARC GTG CGT ACC CCA TGT TTG AGG TGG AA		S	129–160
	CHIKV RR3	TCC AGG ATG GTT GAG TCG GGR TCA ATT TCC T		As	234–264
	CHIKV probe	TGA GTC GGG RTC AAT TTC CTG CTC TAT TAG XT X fT TAT RGC TAG ATG CGA GA-Ph		S	204–253
real-time RT-PCR 1	CHIKV F2	GCA TAY AGG GCY CAT ACM GCA TC	E1 gene	S	10354–10376
	CHIKV R	CAT GRT CGC CGT TTG CAT		AS	10450–10433
	CHIKV TM	Cy5-AAG GAC GCG RAG CTT AGC TGA TGC-BBQ		AS	10401–10378
real-time RT-PCR 2	CHIKV FP1	YGA YCA YGC MGW CAC AG		S	10443–10459
	CHIKV RP1	AAR GGY GGG TAG TCC ATG TT		AS	10549–10568
	CHIKV P2	6FAM -CCA ATG TCY TCM GCC TGG ACR CCK TT-TMR		S	10486–10511

XT, BHQ1-dT; X, Tetrahydrofuran; fT, FAM-dT; Ph, phosphate; Y, C or T; R: A or G; W, A or T; K, G or T; M, A or C; S, original sequence; AS, reverse complementary

### RNA Standards

In-vitro RNA was synthesized using the RiboMAX Large Scale RNA Production System-T7 (Promega, Mannheim, Germany) according to the manufacturer´s instructions and quantified using a Nanodrop ND-1000 spectrophotometer (Thermo Scientific, Dreieich, Germany).

### Real-Time RT-PCR

CHIKV-genomic RNA was tested and quantified by an in-house real-time RT-PCR targeting the E gene using in vitro transcribed RNA standards as described previously [35]. The real-time RT-PCR assays is a CHIKV group-specific assay and was carried out in a one-step format using an ABI 7500 real-time PCR system and the AgPath-ID One-Step RT-PCR Kit. To quantify CHIKV genomic RNA copies, ten-fold serial dilutions of the standard *in vitro* transcribed RNA ranging from 5 to 5 x 10^5^ RNA copies/reaction were tested within the same sample run. The sensitivity of this real-time RT-PCR was determined at nine RNA copies in 95% of the cases by probit regression.

Additionally, a second real-time RT-PCR assay targeting the E gene based on 149 CHIKV sequences was used [36]. It was performed using the LightCycler 480 RNA Master Hydrolysis Probes (Roche, Mannheim, Germany) on the LightCycler 2.0 and the following temperature profile: RT at 63°C for 3 min, activation at 95°C for 30 sec and 45 cycles of 2-steps PCR at 95°C for 5 sec and 60°C for 15 sec. This real-time RT-PCR had an analytical sensitivity of 39 RNA molecules detected by performing the probit analysis.

### CHIKV RT-RPA Assay

The real-time RT-RPA assay was performed in 50 μl reaction volume using the TwistAmp RT exo kit (TwistDx, Cambridge, UK) which provides all enzymes and reagents necessary for the reverse transcription step and DNA amplification in lyophilized pellets according to the manufacturer’s instruction. Briefly, 29.5 μl of rehydration solution were mixed with 7.2 μl of ddH_2_O, 420 nM of each primer and 210 nM of target specific RPA exo-probe. A total of 41.5 μl of this master mix was dispensed into each tube of the eight-tubes strip and 5 μl of RNA template was added to the master mix and mixed. Finally 46.5 μl of master mix/template solution was transferred to each lyophilized RPA pellet of the eight-tubes strip provided in the kit. For each sample, 3.5 μl magnesium acetate (280 mM) was added into the lid of the reaction tubes and the tubes were closed carefully. Through centrifugation of tubes, the magnesium acetate was dropped into each reaction simultaneously to trigger the RT-RPA reaction. The reaction tubes were vortexed, centrifuged and then placed in the ESE Quant Tubescanner (Qiagen Lake Constance GmbH, Stockach, Germany) for real-time monitoring of fluorescence. The reaction was performed at 39°C for 15 minutes, with brief mixing and centrifugation of reaction tubes after four minutes. The resulting amplification curves were analyzed by ESEQuant Tube Scanner software Version 1.0 and threshold values were determined by slope validation i.e. slope (mV/min) values were compared in order to distinguish positive from negative results.

### Analytical Sensitivity

A dilution range between 10^7^ and 10^1^ of the nsP1 RNA molecular standard was used to determine the CHIKV RPA assay sensitivity for two RPA primer pair combinations (RF+RR3 or RF2+RR2, Table 3). A probit regression analysis was performed on eight RPA runs of each combination using STATISTICA (StatSoft, Hamburg, Germany). Additionally, RNA extracts were prepared from 10-fold serial dilutions of two CHIKV strains culture supernatant. RNA extracts containing low viral RNA load were prepared in 5-fold serial dilution to determine the limit of detection (LOD). Aliquots of these RNA extracts were stored at -80°C until further use. The sensitivity of the RF+RR3 combination was evaluated by testing RNA extracts simultaneously on CHIKV RT-RPA and real-time RT-PCR assays in order to compare results and determine the LOD of the RT-RPA assay. Additionally a panel of 18 different CHIKV strains including strains from the current CHIKV outbreaks representing all known three genotypes: Asian, West African and Eastern/Central /South African (ECSA) (Table 1) were tested.

### Assay Cross-Reactivity

In total, twenty-two viruses including eleven *alphaviruses*, ten *flaviviruses* and one *phlebovirus* were used to determine the assay cross reactivity (Table 2).

### Diagnostic Sensitivity and Specificity

Twenty CHIKV positive sera and 58 plasma samples with suspected CHIKV infection were tested by real-time RT-PCR and RT-RPA assays to evaluate diagnostic specificity and sensitivity of the assay. RNA was extracted from these clinical samples using the QIAamp Viral RNA Mini Kit and 5 μl of eluated RNA was tested in each assay.

## Results

### Design and Optimization of RT-RPA

**Fig 1:**
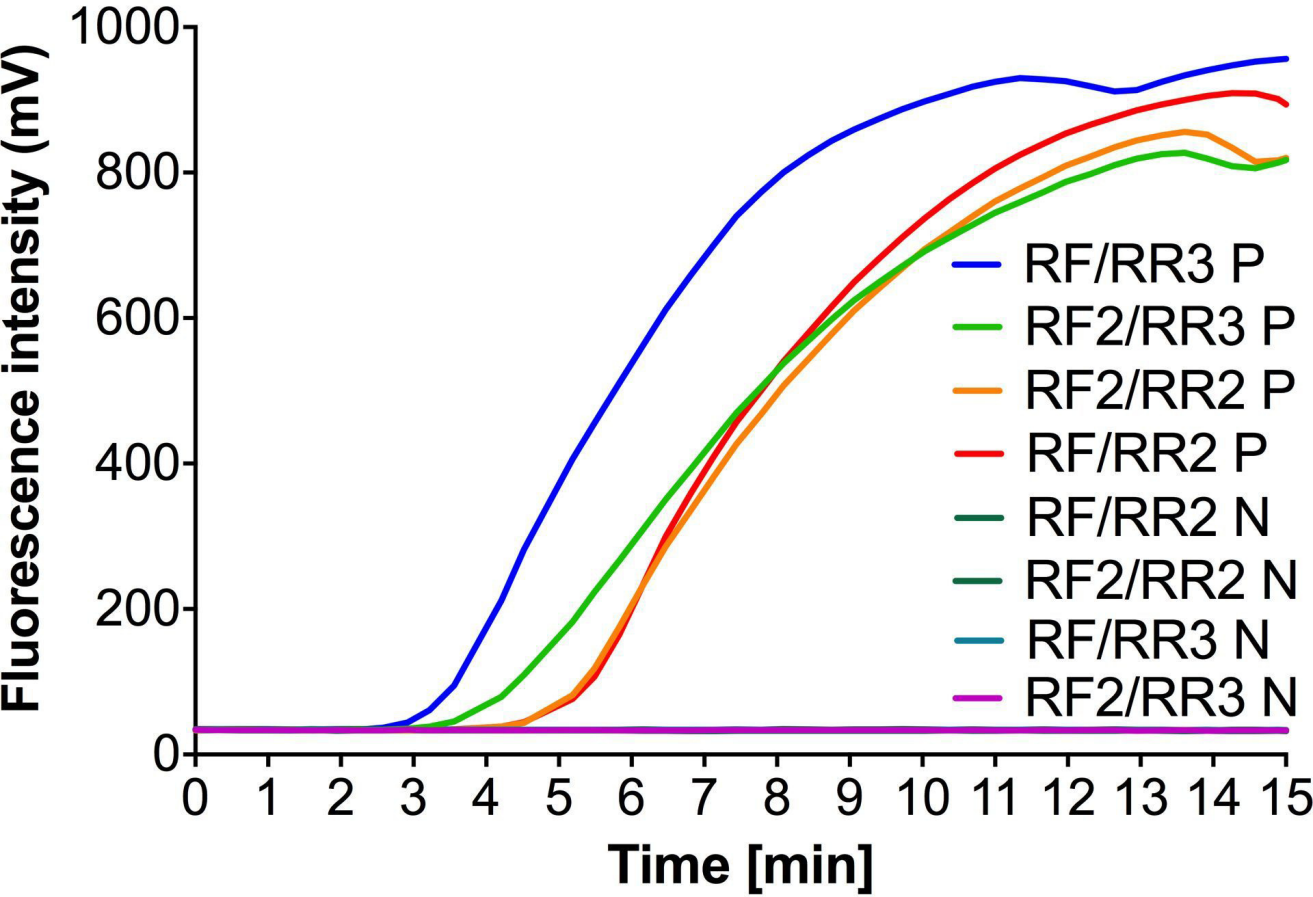
Real-Time RT-RPA run with different combinations of the designed primers. Four primer combinations were tested with the LR isolate (P): RF+RR2, RF2+RR2, RF+RR3 or RF2+RR3. The negative control (N) was water. The RF+RR3 yielded the best RPA fluorescence signal.

For the development of the CHIKV-specific RT-RPA assay, two forward primers, two reverse primers and one RPA probe were designed in the conserved nsP1 target region. To identify the best primer combination, a total of four combinations of were tested on RNA sample of the CHIKV LR strain. Fig 1 illustrates the results of testing the primer combinations and indicates that all four primer combinations were able to amplify CHIKV RNA efficiently within a maximum of six minutes. However, the primer combination CHIKV RF/RR3 resulted in an earlier amplification starting point (after 2.7 minutes) with s higher fluorescence intensity than the other three combinations (Fig 1).

### Analytical Sensitivity of RT-RPA Assay

**Fig 2:**
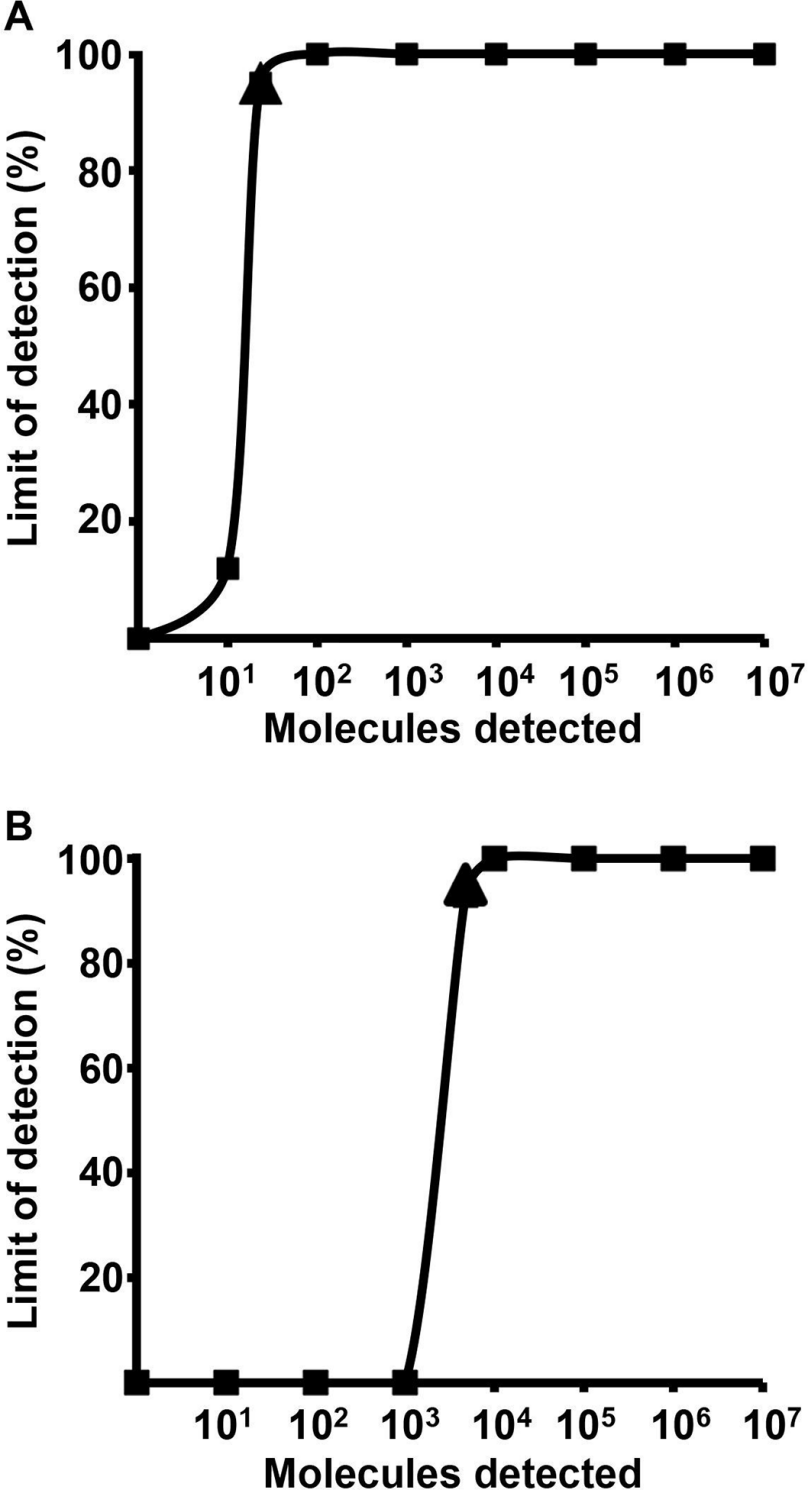
Probit regression analyses of RT-RPA assays using two primer combinations. (A) RF+RR3 and (B) RF2+RR2. The data sets of eight RT-RPA assay runs using serial dilutions of *in vitro* transcribed nsP1 RNA ranging from 10^7^ to 10^1^ RNA molecules were included in the analysis. Triangle sensitivity value at 95% probability.

The analytical sensitivity of both the RF+RR3 and RF2+RR2 combinations were determined using the *in vitro* transcribed RNA standard (n = 8) (S1 Fig). The calculated sensitivity at 95% probability was 23 and 4310 RNA molecules/reaction for RF+RR3 and RF2+RR2, respectively (Fig 2).

**Fig 3:**
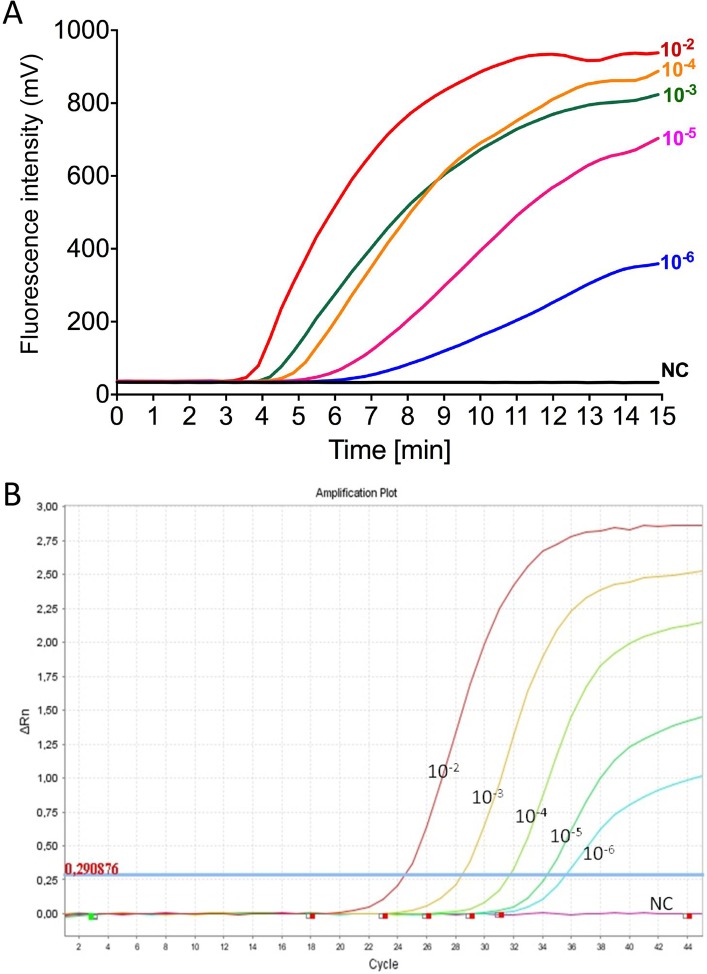
**Comparison between the results of CHIKV RT-RPA RF/RR3 assay (A) and the real-time RT-PCR (B) using 10-fold dilutions of extracted RNA from CHIKV isolate LR.** Both assay amplified down to 80 genome copies (represented by 10^−6^ dilution of the CHIKV).

RNA extracts of a 10-fold serial dilution of two CHIKV strains culture supernatant (LR and IN) were tested by CHIKV RT-RPA using the RF+RR3 primers and real-time PCR to determine the analytical sensitivity. Comparative data are plotted in Fig 3. The results indicated that both the RT-RPA and the real-time PCR detected RNA linearly over 5 log_10_ -steps of a serial dilution of viral RNA. Similar results were also obtained with the CHIKV IN strain (S1 Table). To determine the LOD of the RT-RPA assay more precisely, six replicates of the RNA extracts containing low viral RNA loads as determined with real-time PCR, were diluted in a 5-fold dilution series in the range of the LOD calculated by probit analysis for the RT-RPA using in vitro transcribed RNA.

Tested by RT-RPA, 6 of 6 (100%) RT-RPA replicates were amplified at a dilution 10^−5^ containing 361 and 467 RNA genome equivalents (GC)/reaction (rxn) for the LR and the IN strain, respectively. At the dilution of 10^−6^ containing 80 and 92 GC/rxn for the LR and the IN strain, respectively, 5 of 6 replicates were amplified by RT-RPA assay. At the lowest dilution of 10^−7^ containing 10 GC/rxn only 2 of 6 and 3 of 6 replicates were detected for both the LR and the IN strain. Overall the probit detection limit in 95% of cases for real-time RT-PCR was 10 GC/rxn, whereas the LOD of RT-RPA was 80 GC/rxn.

### Analytical Specificity and Cross-Reactivity of the RT-RPA Assay

The sensitivity of the RT-RPA assay was assessed by testing 18 CHIKV strains representing all three genotype (Table 1), the specificity was tested with eleven different *alphaviruses*, ten *flaviviruses* and one *phlebovirus* (Table 2). The CHIKV RT-RPA assay utilizing RF+RR3 efficiently detected all 18 CHIKV strains and the O'nyong'nyong virus (ONNV), while RT-RPA assay using RF2+RR2 amplified only the CHIKV strains. There was no cross reactivity found to other viruses of the cross reactivity panel (Table 2 and S2 Fig).

### Performance of the RT-RPA Assay on Clinical Samples

The diagnostic sensitivity and specificity of the CHIKV RT-RPA assay was assessed with plasma samples from 58 suspect CF cases and compared to two CHIKV specific real-time RT-PCR tests during a field trial in Bangkok, Thailand. Both CHIKV real-time RT-PCR detected 36 out of 58 sample (62%) positive. The Ct values obtained by real-time RT-PCR for positive samples ranged from 20.19 to 36.02 (1.6x10^4^ -1x10^8^ GC/rxn). In comparison to real-time RT-PCR, RT-RPA correctly identified all 36 positive samples and did not detect the 22 negative samples with 100% sensitivity and specificity (PPV: 1, NPV: 1, Fig 4). Additionally, we tested 20 sera from acute CHIK patients from France. All three methods efficiently detected 20 out of 20 CHIKV positive samples.

**Fig 4:**
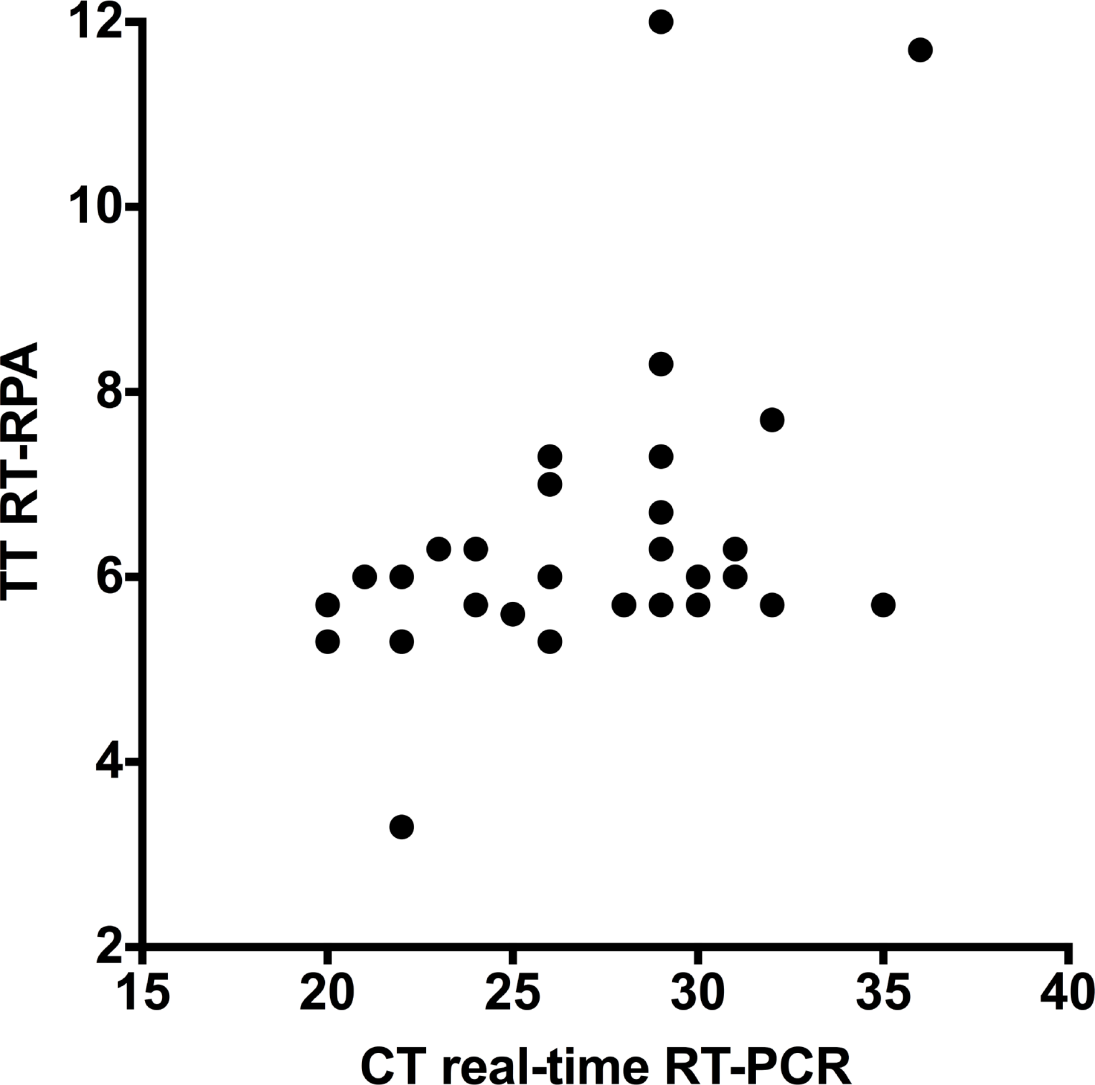
Results of screening clinical samples using real-time RT-RPA and RT-PCR assays. Thirty-six samples were positive in both assays with 100% agreement. Twenty-nine dots are shown as seven spots overlap. No correlation was found between RT-PCR and RT-RPA values (R = 0.2).

## Discussion

CHIKV is treated symptomatically by use of painkillers and anti-inflammatory drugs. As there is no effective specific antiviral drug and currently only experimental CHIKV vaccine development. Laboratory diagnosis of CHIKV is very important for effective outbreak management including clinical management and vector control. Therefore, there is a need for a reliable, rapid and portable diagnostic method, which can be deployed in the field during outbreaks.

In this study, we developed a RT-RPA assay for rapid detection of CHIKV in clinical samples, which can be easily deployed to rural health care centers or used in field investigations. We designed a highly sensitive set of RPA primers and exo-probe, which detect CHIKV down to 80 GC/rxn. This detection limit is slightly higher than the 10 GC/rxn detected by reference real-time RT-PCR methods. However, the sensitivity of RT-RPA was sufficient to detect CHIKV infection in clinical samples from Thailand and France with an overall sensitivity and specificity of 100% in comparison to real-time RT-PCR. Previous studies described high titer viraemia exceeding 10^9^ GC/ml in the acute phase of CF [37,38]. In our study of 20 CF acute samples, viral load ranged from 1.6x10^6^ -1x10^10^ GC/ml (1.6x10^4^ -1x10^8^ GC/rxn).

The RT-RPA assay detected a panel of 18 different CHIKV strains of all known three genotypes. There was no cross-reactivity of the RT-RPA assay with tested common alpha- and arboviruses except for ONNV detected with the primer combination (RF+RR3), while the combination RF2+RR2 did not detect the ONNV (S2 Fig). Cross reactivity of CHIKV RT-RPA assay to ONNV could be due to 85% similarity of the genomes of ONNV to CHIKV.

RT-RPA primers RF/RR3 harbour four mismatches in RF and seven mismatches in RR3 to the ONNV sequences (S3 Fig). This seems to reflect the fact that RPA assays have been shown to amplify target genes in the presence of up to nine mismatches [25,30]. Moreover, the position of the mismatch did not influence the amplification step as in the real-time PCR [29]. However, we chose primer pair RF/RR3 due to its faster amplification (Tt: 3.3) and highly analytical sensitivity (23 RNA copies) in comparison to the more specific primer pair RF2/RR2 (Tt: 3.7 and analytical sensitivity: 4310). If necessary, the latter can be used for the differentiation between CHIKV and ONNV e.g. when used in ONNV endemic regions of Africa whereas the former can be used in Asia, Europe and the Americas without any issue.

The RT-RPA assay using RF/RR3 demonstrated a sensitivity of 100% on CHIKV samples of the recent external quality assessment (EQA) study for molecular diagnosis but also detected one sample containing ONNV. Both real-time RT-PCR tests used in this study showed no cross reactivity to ONNV which indicates superior specificity compared to commercial PCR methods tested in the EQA study, in which 46.2% showed cross reactivity to ONNV [39].

Two loop-mediated isothermal amplification (LAMP) assays for the detection of CHIKV have been published [40,41]. The LAMP assay require at least four primers and six binding sites and results can be usually observed visually after 30–60 minutes [42]. Recent LAMP assay developments begin to show shorter run times [43]. In contrast, the RPA assay is much faster (3–15 minutes) and utilized two primers and one probe i.e. three binding sites. In addition, RPA reagents are provided in a lyophilized pellet stable at ambient temperature (25–38°C) [26,28,33], which allow independence from the cooling chain.

The RPA is a promising technology to perform molecular assay at point of need. Nevertheless, the RPA primer and probe design is still challenging, as dozens of primers combinations must be tested in order to select a functional one. The current RPA assay protocol requires four pipetting steps, which is still less than the real-time PCR method but further development to miniaturize the assay is required. The cost of test per sample is around 5 USD. Lowering the cost to 1 USD will maximum its use in the affected countries.

The CHIKV RPA assay presented here is a promising tool for CHIKV diagnostics at the point of need. Integration into a multimer or multiplex assay for simultaneous and differential detection of CHIKV, Dengue virus and Zika virus as well as an internal positive control would improve outbreak investigations, since the three viruses induce the same clinical picture upon infection and increasingly co-circulate in many parts of the world. Furthermore, combination with a simple extraction method for allowing isolation of virus or virus-infected cells from the whole blood and simple lysis protocol will maximize its employment at low resource settings.

## Supporting Information

S1 Fig Reproducibility of CHIKV RPA assays employing the two primers combinations (A, RF+RR3; B, RF2+RR2). The RPA assays were conducted eight times using 10-fold serial dilution of the RNA molecular standards. CHIKV RPA assay produced results between 2 to 12 minutes. 10^7^−10^4^ RNA molecules were detected 8 out of 8 runs applying both primers combinations. RF+RR3 primers amplified 10^3^−10^2^ copies 8 out of 8 times, while 10^1^, one out of eight times. 10^3^−10^1^ RNA copies were not identified by the CHIKV RF2+RR2 RPA assay. The error bars represent the rang.
(PDF)

S2 Fig Results of testing ChikV (black) and ONNV (Red) samples with RT-RPA assays with two primer pair combinations, A) RF+RR3 and (B) RF2+RR2. The RF+RR3 assay is more sensitive but is able to amplify the ONNV gene, while the RF+RR3 is less sensitive but did not detect the ONNV.
(PDF)

S3 Fig Alignment of Chikungunya RT-RPA primers and exo-probe sequences with the Chikungunya and O'nyong'nyong viruses sequence using GENEIOUS. (PDF)

S1 Table Determining the sensitivity of RT-RPA assay. Ten-fold serial dilutions of ChiKV isolates LR and In, were tested in both real-time RT-PCR and RT-RPA.
(PDF)

S1 Checklist (PDF)

S1 Flowchart A diagram represents the workflow of screening clinical samples using real-time RT-PCR and RT-RPA assays. (PDF)

S1 Approval Ethical approval from the Mahidol University Institutional Review Board on the use of the samples from suspected CHIKV-infected cases. (PDF)
