# Supplementary material for: A Field-Deployable Reverse Transcription Recombinase Polymerase Amplification Assay for Rapid Detection of the Chikungunya Virus
Source: PLoS Negl Trop Dis. 2016 Sep 29;10(9):e0004953. doi: 10.1371/journal.pntd.0004953 (PMC5042537; doi:10.1371/journal.pntd.0004953)
Supplement: S1 Fig — Reproducibility of CHIKV RPA assays employing the two primers combinations (A, RF+RR3; B, RF2+RR2). The RPA assays were conducted eight times using 10-fold serial dilution of the RNA molecular standards. CHIKV RPA assay produced results between 2 to 12 minutes. 107−104 RNA molecules were detected 8 out of 8 runs applying both primers combinations. RF+RR3 primers amplified 103−102 copies 8 out of 8 times, while 101, one out of eight times. 103−101 RNA copies were not identified by the CHIKV RF2+RR2 RPA assay. The error bars represent the rang. (PDF) [file pntd.0004953.s001.pdf]

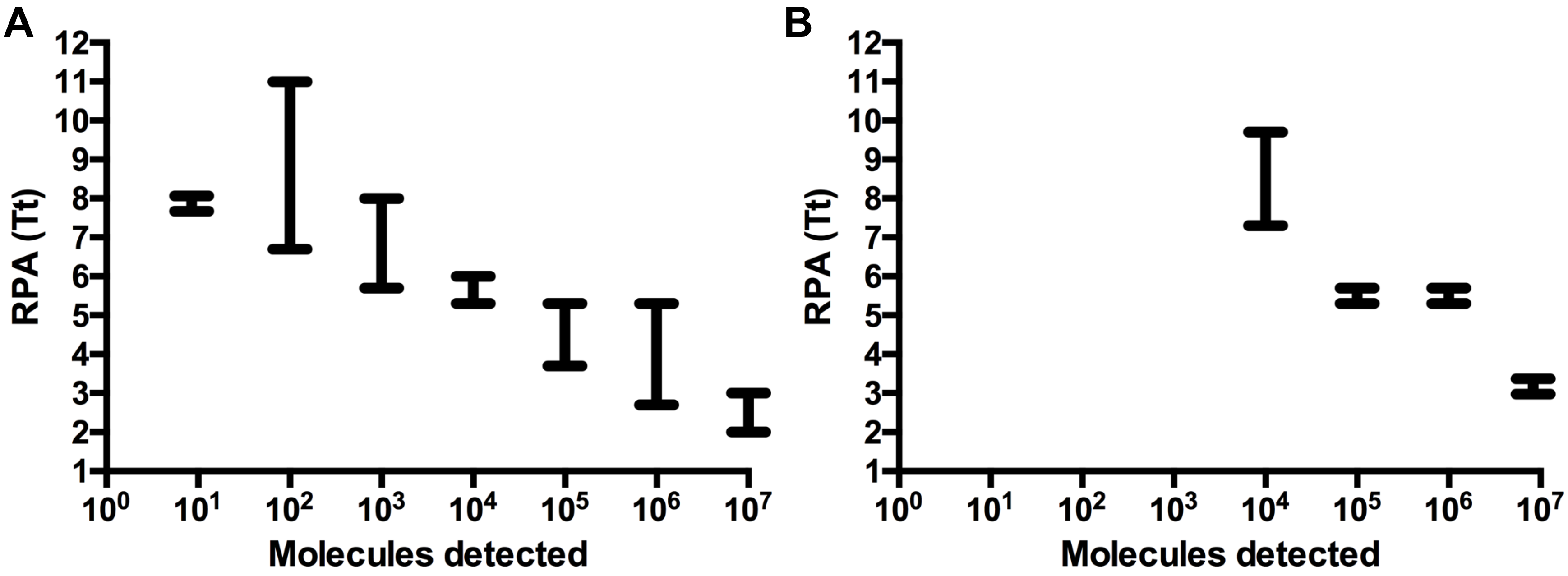

S1 Fig. Reproducibility of CHIKV RPA assays employing the two primers combinations (A, RF +RR3; B, RF2+RR2). The RPA assays were conducted eight times using 10-fold serial dilution of the RNA molecular standards. CHIKV RPA assay produced results between 2 to 12 minutes. 10^7-10^4 RNA molecules were detected 8 out of 8 runs applying both primers combinations. RF +RR3 primers amplified 10^3-10^2 copies 8 out of 8 times, while 10^1, one out of eight times. 10^3-10^1 RNA copies were not identified by the CHIKV RF2+RR2 RPA assay. The error bars represent the rang.
