# Supplementary material for: A Field-Deployable Reverse Transcription Recombinase Polymerase Amplification Assay for Rapid Detection of the Chikungunya Virus
Source: PLoS Negl Trop Dis. 2016 Sep 29;10(9):e0004953. doi: 10.1371/journal.pntd.0004953 (PMC5042537; doi:10.1371/journal.pntd.0004953)
Supplement: S2 Fig — Results of testing ChikV (black) and ONNV (Red) samples with RT-RPA assays with two primer pair combinations, A) RF+RR3 and (B) RF2+RR2. The RF+RR3 assay is more sensitive but is able to amplify the ONNV gene, while the RF+RR3 is less sensitive but did not detect the ONNV. (PDF) [file pntd.0004953.s002.pdf]

A

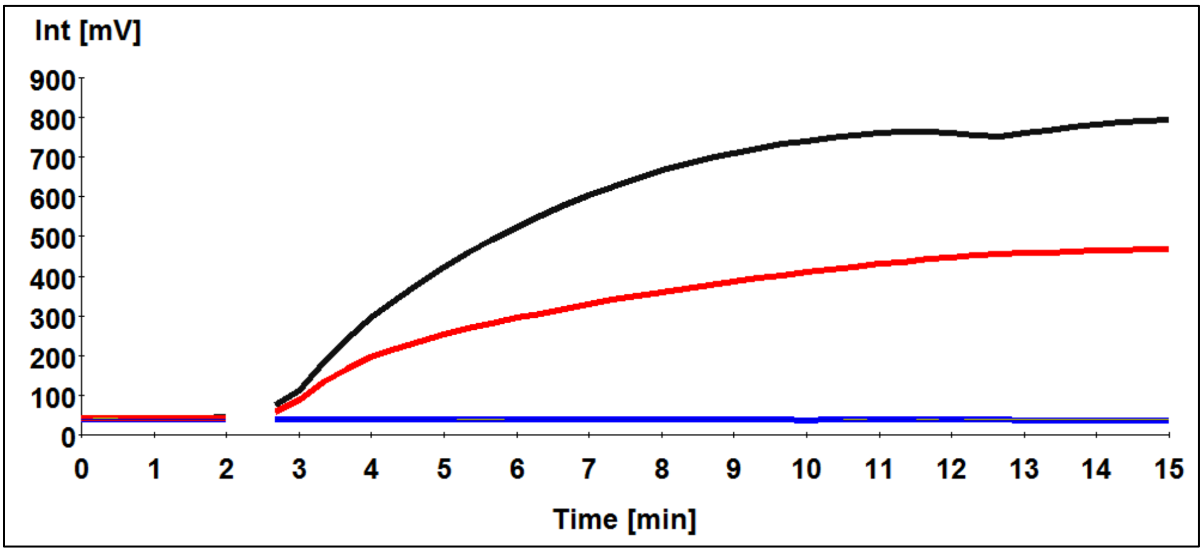

B

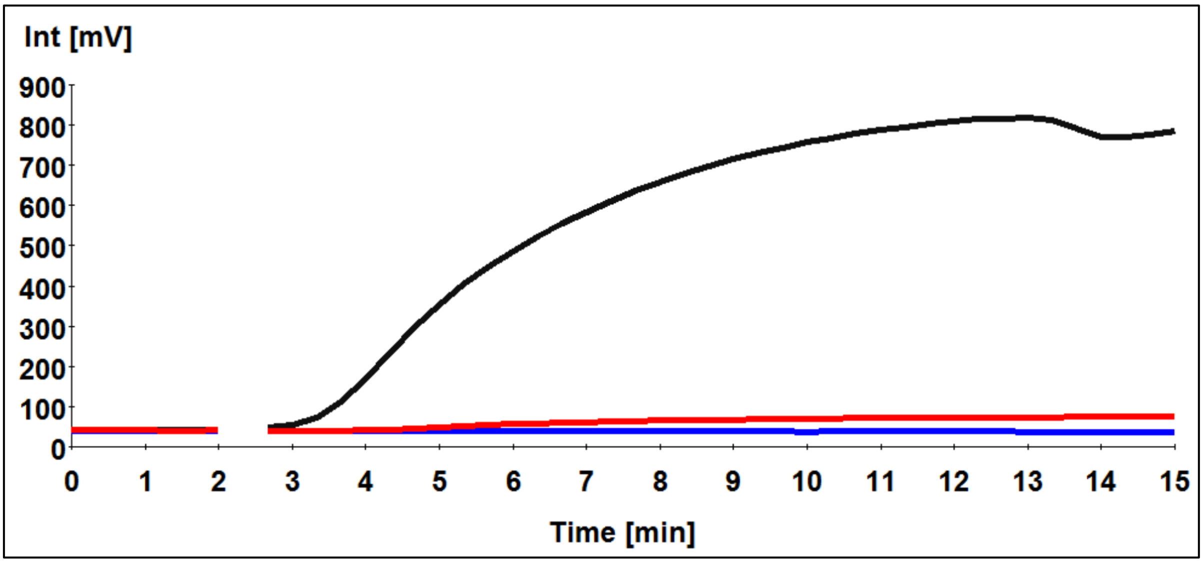

S2 Fig. Results of testing ChikV (black) and ONNV (Red) samples with RT-RPA assays with two primer pair combinations, A) RF+RR3 and (B) RF2+RR2. The RF +RR3 assay is more sensitive but is able to amplify the ONNV gene, while the RF +RR3 is less sensitive but did not detect the ONNV.
