# Supplementary material for: A Field-Deployable Reverse Transcription Recombinase Polymerase Amplification Assay for Rapid Detection of the Chikungunya Virus
Source: PLoS Negl Trop Dis. 2016 Sep 29;10(9):e0004953. doi: 10.1371/journal.pntd.0004953 (PMC5042537; doi:10.1371/journal.pntd.0004953)
Supplement: S3 Fig — (PDF) [file pntd.0004953.s003.pdf]

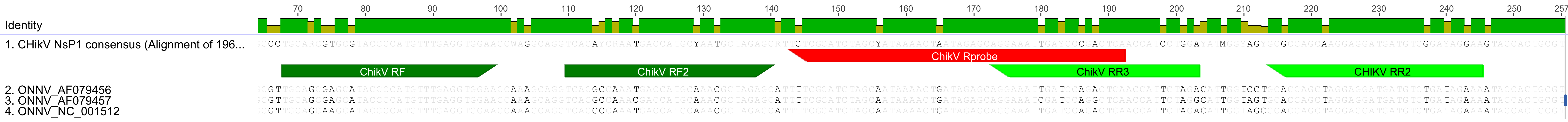

S3 Fig. Alignment of Chikungunya RT-RPA primers and exo-probe sequences with the Chikungunya and O'nyong'nyong viruses sequence using GENEIOUS.
