# Supplementary material for: A Field-Deployable Reverse Transcription Recombinase Polymerase Amplification Assay for Rapid Detection of the Chikungunya Virus
Source: PLoS Negl Trop Dis. 2016 Sep 29;10(9):e0004953. doi: 10.1371/journal.pntd.0004953 (PMC5042537; doi:10.1371/journal.pntd.0004953)
Supplement: S1 Table — Ten-fold serial dilutions of ChiKV isolates LR and In, were tested in both real-time RT-PCR and RT-RPA. (PDF) [file pntd.0004953.s004.pdf]

S1 Table. Determining the sensitivity of RT-RPA assay. Ten-fold serial dilutions of ChiKV isolates LR and In, were tested in both real-time RT-PCR and RT-RPA.

**Dilutions**	**In strain**		**LR strain**	
	**Real-time PCR (Ct)**	**RT-RPA (Tt)**	**Real-time PCR (Ct)**	**RT-RPA (Tt)**
10^{-2}	24.69	4	24.01	3.7
10^{-3}	28.08	4.7	27.91	4.3
10^{-4}	31.4	5	31.22	4.7
10^{-5}	34.36	6	33.76	5.3
10^{-6}	34.38	5.3	34.94	6.3

Ct: Cycle Threshold value of real-time RT-PCR; Tt: TimeThreshold value of RT-RPA in minutes.
