# Supplementary material for: A Field-Deployable Reverse Transcription Recombinase Polymerase Amplification Assay for Rapid Detection of the Chikungunya Virus
Source: PLoS Negl Trop Dis. 2016 Sep 29;10(9):e0004953. doi: 10.1371/journal.pntd.0004953 (PMC5042537; doi:10.1371/journal.pntd.0004953)

**flow of participants through the study: A field-deployable reverse transcription recombinase polymerase amplification assay for rapid detection of the Chikungunya virus**

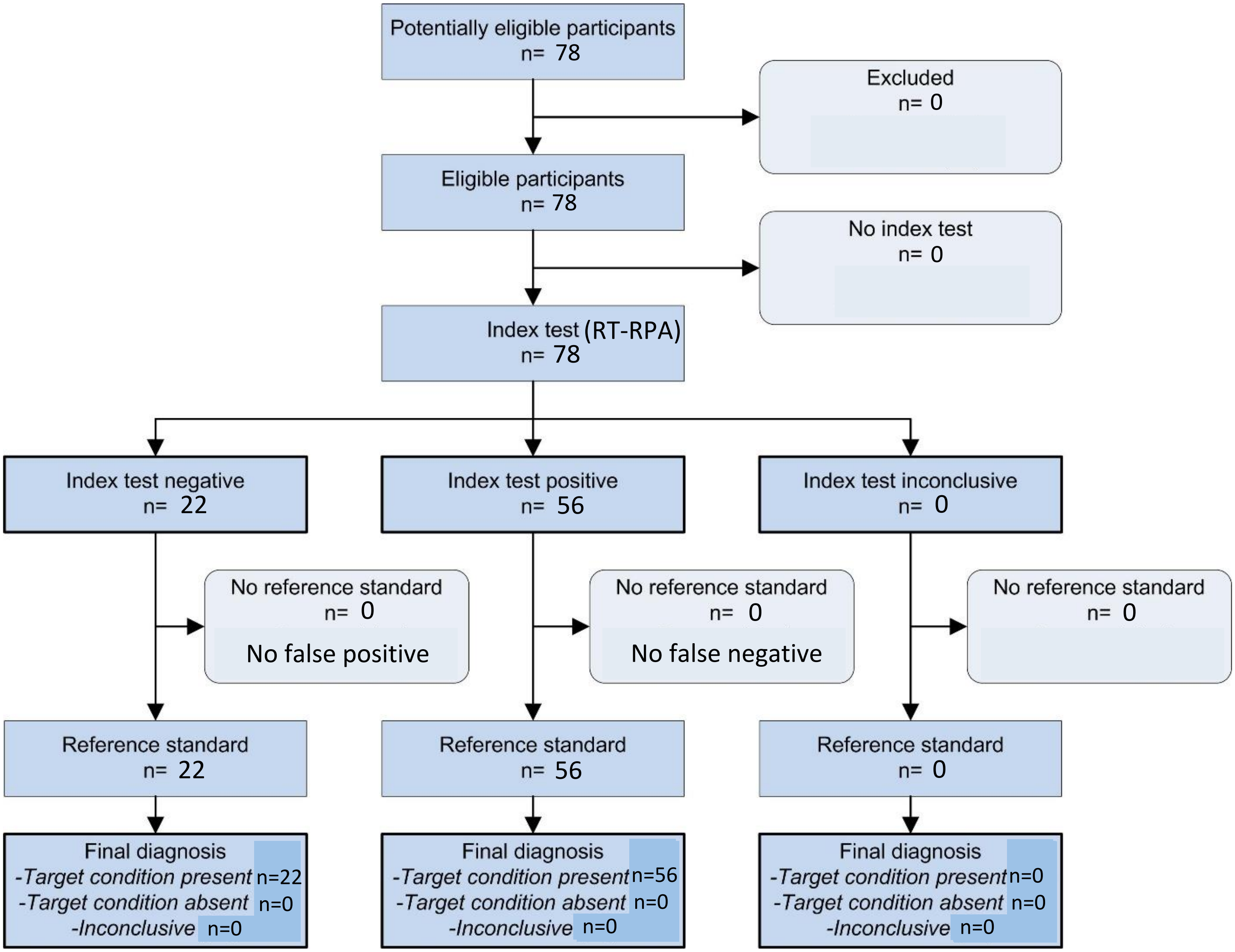

Supplement: S1 Flowchart — (PDF) [file pntd.0004953.s006.pdf]
