# Supplementary material for: A Field-Deployable Reverse Transcription Recombinase Polymerase Amplification Assay for Rapid Detection of the Chikungunya Virus
Source: PLoS Negl Trop Dis. 2016 Sep 29;10(9):e0004953. doi: 10.1371/journal.pntd.0004953 (PMC5042537; doi:10.1371/journal.pntd.0004953)
Supplement: S1 Approval — (PDF) [file pntd.0004953.s007.pdf]

COA. No. MU-IRB 2010/251.3108

Documentary Proof of Mahidol University Institutional Review Board

Title of Project: Host and Viral Determinants in Chikungunya Virus-infected

Principal Investigator: Associate Professor Dr. Sukathida Ubol

Co-investigators: Mr.Weerawat Phuklia
Miss Jindarat Lohachanakul

Name of Institution: Faculty of Science

Approval includes:
1) MU-IRB Submission form version received date 31 August 2010
2) Participant Information Sheet version date 26 August 2010
3) Informed Consent form version date 29 June 2010

Mahidol University Institutional Review Board is in full compliance with International Guidelines for Human Research Protection such as Declaration of Helsinki, The Belmont Report, CIOMS Guidelines and the International Conference on Harmonization in Good Clinical Practice (ICH-GCP)

Date of Approval: 31 August 2010

Date of Expiration: 30 August 2011

Signature of Chairman: ....
(Professor Shusee Visalyaputra)

Signature of Head of the Institute: ....
(Associate Professor Sansanee Chaiyaroj)
Vice President for Research and Academic Affairs

Office of the President, Mahidol University, 999 Phuttamonthon 4 Rd., Salaya, Phuttamonthon District, Nakhon Pathom 73170. Tel. (662) 8496223-5 Fax. (662) 8496223
